# PD210 Sustainable Innovation In UK Care Homes: Acoustic Monitoring Systems In A “Living Lab” Setting - Technology Assessment Example

**DOI:** 10.1017/S026646232400432X

**Published:** 2025-01-07

**Authors:** Quang A Nguyen, Petra A Wark, Louise McCabe, Dingchang Zheng, Yanguo Jing, Mark Collinson, Toshio Nomura, Vic Rayner, Ed Russell, Ala Szczepura

## Abstract

**Introduction:**

Successful development of technologies to support older people in care homes remains challenging. The strategic establishment of a group of residential settings providing 24-7 living labs plus an innovation hub is described. Acoustic monitoring (AM) for continuous nighttime checking of residents was selected for assessment. A pilot study in 2016 identified potential cost savings, reduced nighttime falls, and improved daytime well-being.

**Methods:**

The AM system was systematically assessed in four WCS Care Group Ltd care homes following demonstration and testing in an innovation hub. The first author undertook a mixed-methods study (2019 to 2023) to assess the technology in terms of falls prevention, practical implementation, and future benefits of enhanced sound classification using artificial intelligence (AI). In 2020, a network of care providers, the National Care Forum (NCF), was funded by National Health Service Digital to develop three similar innovation hubs across England. The NCF undertook a national survey to identify priorities in terms of implementing new technologies in care homes.

**Results:**

Structured interviews with care workers and observational assessment in four care homes confirmed that the AM system has the potential to identify active residents and reduce the risk of falls, although it cannot offer fall prediction or prevention functionality. Two new functions were proposed. Eight machine learning models for sound classification and demonstration tests in three simulated settings supported feasibility and adoption. A post-demonstration survey (n=39) identified a high probability of adoption for these additional functions. The NCF survey found that the top four new technologies of interest to care homes were medication management systems, electronic care planning, wearable GPS trackers, and AM systems.

**Conclusions:**

Care homes report interest in procuring AM systems, which could reduce the risk of falls. In addition, new AI-supported functions are acceptable to care providers. The UK government has proposed that 20 percent of care homes introduce AM by March 2024. Guidelines for future assessment of sustainable care technologies in living lab settings now need to be developed.

